# Hybrid simulation and immersive, lived-experience perspectives to shape medical student attitudes towards patients experiencing emotional distress, suicidality, and self-harm

**DOI:** 10.1186/s41077-025-00336-4

**Published:** 2025-03-24

**Authors:** Ellen Davies, Natalie Mills, Adam Montagu, Anna Chur-Hansen, Scott Clark

**Affiliations:** 1 Adelaide Health Simulation, Faculty of Health and Medical Sciences, The University of Adelaide, Level 2, AHMS Building, 4 North Terrace, Adelaide, SA 5005 Australia; 2 Adelaide Medical School, Faculty of Health and Medical Sciences, The University of Adelaide, Adelaide, Australia; 3 School of Psychology, Faculty of Health and Medical Sciences, The University of Adelaide, Adelaide, Australia

**Keywords:** Health simulation, Psychiatry, Virtual reality, Simulated patients, Trauma-informed care, Immersive simulation, Stigma, Attitudes

## Abstract

**Background:**

When medical students enter their first psychiatry rotation, they often feel under-prepared for the complex milieu of psychopathology, emotional distress, and complex psychosocial issues. Simulation is valued for its ability to orient learners to new environments. In this project, a hybrid simulation workshop was designed and delivered for fourth-year medical students. This study aimed to examine students’ experience of this workshop and to explore participant attitudes towards people who experience mental distress.

**Methods:**

Fourth-year undergraduate medical students were invited to complete pre- and post-workshop questionnaires that contained a series of previously developed scales, including the Stigma of Suicide Scale, the Literacy of Suicide Scale, the General Help-Seeking Behaviour Scale, the Attitudes and Confidence in the Integration of Psychiatry Scale, and the Satisfaction with Simulation Experience Scale.

**Results:**

From a cohort of 172, 118 students participated (68.8%). The mean percentage of suicide literacy rose from 65.8 to 70.1%, with the highest literacy in the “treatment and management” domain (pre-workshop mean 92.9%, post-mean 95.0%) and lowest in the “signs and symptoms” domain (pre-workshop mean 38.0%; post-mean 44.5%). Suicide stigma was low both pre- and post-workshop. In both pre- and post-workshop, participants identified feeling most confident about screening for depression and least confident about managing symptoms of anxiety for patients and their relatives. Concerningly, 11% of the cohort stated they would not seek help themselves if they experienced thoughts of self-harm or suicide.

**Conclusion:**

Using a combination of simulation modalities, students were oriented to their psychiatry placements. Importantly, this orientation focused on the experiences of people with lived experience of mental illness and how health professionals impact patient journeys through health and mental health services. Findings suggest this type of simulation workshop can support students in their dispositional readiness for placement in psychiatry units.

**Supplementary Information:**

The online version contains supplementary material available at 10.1186/s41077-025-00336-4.

## Introduction

Attitudes towards suicide, self-harm, and people who experience mental illness are shaped through numerous channels, including social and traditional media, culture, personal and proximal experiences, and social contexts [30]. These attitudes will often determine how health professionals choose to engage (or not) with people experiencing mental distress [30]. Medical practitioners across a range of fields will encounter people with current and previous histories of self-harm behaviors and people who have thought about or attempted to end their lives [7, 30]. As emerging health professionals, medical students require knowledge, skills, and tools to engage with people who are experiencing mental distress. Conversations about these topics with patients, relatives, and the broader community can be perceived as difficult. Even trained clinicians can lack the confidence to engage with people who have experienced this degree of mental and emotional distress, resulting in avoidance behaviors and the perpetuation of mental health stigma [18, 26].

Influencing and shaping medical students’ attitudes, values, comprehension, and sense of confidence to work with people experiencing mental illness requires various complementary strategies. The presentation of evidence, the early integration of psychiatric content in the broader medical curricula, and access to experts in the field have all been identified as important interventions for improving attitudes and understanding of suicide, self-harm behaviors, and mental illness [15].

Clinical placement in mental health units has traditionally been a vehicle for exposing students to psychiatry as a field. While being in a work environment provides significant learning opportunities [6], students’ readiness for entering mental health units needs to be carefully considered. A 2014 Australian study investigating suicide literacy, suicide stigma, and attitudes towards and intentions regarding help-seeking behaviors in medical students found that increased clinical experience improved knowledge about suicidality but reduced the likelihood of personal help-seeking [10]. The authors concluded that more needed to be done for medical students than simply facilitating exposure to people presenting with self-harm and suicidality while on clinical placement. They advocated for increased interventions to improve mental health literacy to reduce stigma towards those with mental ill-health and those who self-harm [10].

Readiness for practice “comprises what individuals know, can do, and value” [5], p. 1). Even if students are prepared conceptually (e.g., have a clinical understanding of mental illness) [5] and are prepared procedurally (e.g., know and have the ability to undertake tasks) [5], consideration for dispositional readiness is necessary. If student attitudes towards people with mental illness are shaped by factors that promote stigma and poor understanding of causes, treatment, and management of mental illness then interventions should be considered to support better attitudinal alignment with recovery-oriented and trauma-informed principles of care [12, 23].

We sought to explore and enhance students’ readiness for clinical placement through a hybrid simulation workshop. Health simulation presents opportunities to explore, practice, and reflect on clinical abilities, confidence, attitudes, knowledge, and skill gaps [25]. Of particular note, simulation affords participants with opportunities to experience uncomfortable situations, such as practicing difficult conversations, in a relatively safe physical and psychological environment [16, 25, 29]. Not only can this environment be safer for healthcare students and professionals, but it can also offer a unique space for people with lived experience of mental illness to have a voice through the representation of their stories [14, 33]. Immersive simulation technologies have recently shown promise for the teaching of skills in mental health care [9, 27]. They have been trialed as tools to enhance emotional engagement, allowing learners to explore their own emotional responses and understanding of new contexts [1, 9, 27, 28].

In this project, we combined lived experience stories presented in an Igloo 360° immersion room, 360° virtual reality (VR) scenarios, and a simulated patient (SP) scenario to form a hybrid simulation workshop that was purposefully linked to students’ 6-week psychiatry rotation (the rotation incorporates both university teaching time and clinical placement). In this study, we aimed to (1) explore fourth-year undergraduate medical students’ attitudes towards psychiatry; (2) evaluate the suicide literacy, suicide stigma, help-seeking intentions, and confidence of this population before and after attending an immersive simulation workshop; (3) explore this population’s self-perceived confidence when engaging with people who have experienced a mental illness, self-harm behaviors, and thoughts of suicide or attempted suicide. The research questions addressed include “how do students perceive and experience their first psychiatric placement?” and “what is the current state of suicide literacy, suicide stigma, and help-seeking intentions in an Australian cohort of undergraduate medical students and do these alter after attending an immersive simulation workshop?”.

A multi-methods project was designed to capture data from questionnaires that have demonstrated validity in previous studies and semi-structured interviews. Data from the semi-structured interviews are reported elsewhere and address the first aim of the project: to explore students’ attitudes towards and experiences related to the psychiatry rotation. This paper outlines the methods and findings from the quantitative data, where students’ suicide literacy, stigma, help-seeking intentions, and confidence are analyzed.

## Methods

### Study design

A hybrid simulation workshop was designed to explicitly meet four learning outcomes for The University of Adelaide (UoA) fourth-year medical students in the week prior to attending their clinical placement in a mental health unit (see Table 1). Pre- and post-workshop surveys that have been used with a previous cohort of Australian undergraduate fourth-year medical students [10] were administered to examine suicide literacy, suicide stigma, help-seeing intentions, and confidence in this single-site cohort before and after attending the workshop.

**Table 1:** Overview of workshop

Station	Description	Intended learning outcome (ILO)
1. LELAN video in a 360° immersion room and psychiatrist-led discussion	The “Care not Treatment” video produced by LELAN was edited for viewing in a 360° immersion room with permission^a^ A discussion about the content of the video was guided by a consultant psychiatrist at the conclusion of the video	ILO1: Describe in detail the impact that healthcare professionals have on patient progress through health systems
2. 360° Virtual Reality (VR) immersive experiences (*x*^2^) with psychiatrist-led discussion	Using VR headsets, students viewed two contrasting versions of a distressed patient’s journey from home to a hospital emergency department. The patient is attended to by ambulance officers after a self-harm incident (superficial cutting to the upper leg). In the first version, staff are disrespectful and do not address the patient’s concerns, compounding distress, and resulting in a behavioral escalation in the hospital. In debriefing, students are encouraged to reflect on the patient experience and how the interaction could be improved. Students are then shown a version with optimized, patient-centered, and trauma-informed care, and the contrast is discussed in debriefing	ILO2: Describe in detail the impact that healthcare professionals have on patient progress through health systems ILO3: Describe the roles and responsibilities of different members of the healthcare team in managing patients exhibiting behaviors of concern
3. Simulated patient (SP) interaction and debrief	The SP from the 360° VR films was central to Station 3. In this station, a pair of students are invited to interview the SP at the point in their journey when they have been in the Emergency Department for 4 h. Students were expected to undertake a structured assessment and to explore safety and risk with the SP Feedback from the SP, tutor, and observers was encouraged and respectfully provided in a discussion and debrief after the simulation scenario concluded	ILO4: Demonstrate confidence and capacity to engage with people with self-harm behaviors, suicidal ideation, or who have attempted suicide ILO5: Demonstrate a structured assessment of a patient presenting with signs and symptoms of a mental illness and present an accurate handover and risk assessment to a colleague

Abbreviations: *LELAN *Lived Experience Leadership & Advocacy Network

^a^https://www.youtube.com/watch?v=dCYfEte_EvY

### Setting

The study was undertaken at UoA, South Australia, with the simulation workshop hosted at Adelaide Health Simulation (AHS)’s Helen Mayo South site. This site is equipped with 10 individual clinical simulation rooms, two large debriefing rooms, and an Igloo 360° immersion room. Hosted at this site are eight HTC Vive headsets and two HTC Vive Pro headsets.

UoA offers a 6-year undergraduate medical program. The majority of applicants to the program are secondary school leavers, thus are around 18 years of age in the first year of study. Students undertake clinical placements across various areas of medicine across 6 years, including for the specialty of psychiatry. In the fourth year of their medical degree UoA medical students undertake six specialty rotations. One of these rotations is “psychiatry,” and clinical placement is undertaken in a mental health unit (for example, an inpatient or community, older persons, adult, or child/adolescent mental health unit). The first week of the psychiatry rotation is traditionally spent attending tutorials and lectures, followed by 5 weeks of clinical placement in mental health units and supervised by psychiatry registrars or consultants. In 2023, the hybrid simulation workshop described in this paper was embedded at the end of the first clinical placement week.

### Participants

All fourth-year medical students from the 2023 (*n* = 172) cohort were invited to participate in online questionnaires pre- and post-simulation workshops. Students were recruited at the commencement of their psychiatry rotation via an announcement in the university’s electronic learning platform and via email. These communications included links to the online surveys. Students were reminded of the study verbally during the workshop orientation and QR codes that linked to the survey were provided onsite at the simulation workshop to maximize recruitment.

### Simulation workshop overview

A half-day, hybrid simulation workshop was embedded into the first week of the psychiatry rotation. The workshop included two stations with immersive technologies (Igloo 360° and VR headset scenarios) and one station with an SP. The workshop was designed to accommodate the 25–30 students who were allocated to each psychiatry rotation (six rotations per year). Groups of ten (or fewer) students rotated across three stations, each led by a consultant psychiatrist (see Table 1). Additional information about this workshop can be found in Supplementary file 1. A summary of approximate costs to conduct the workshops is provided in Supplementary file 3.

### Data sources and measurement

Data were collected via a suite of questionnaires that have demonstrated validity in previous studies with populations who share similar demographics. These were intended to measure suicide stigma, suicide literacy, help-seeking behavior, confidence relating to practice in psychiatry, and satisfaction with the simulated workshop. Participants were asked to provide basic demographic data, including age, gender, country of birth, and languages spoken.

The 27-item Literacy of Suicide Scale (LOSS), as published by Chan et al. [10] and further described by Calear et al. [8], was used to explore participants’ mental health literacy. This scale assesses participants’ understanding of suicide in what the authors’ term “suicide literacy,” in four domains of knowledge: signs and symptoms, causes or the nature of suicidality, risk factors, and treatment and prevention [8]. Participants have the option to answer “true,” “false,” or “I don’t know” and are scored as either correct or incorrect/unsure. The LOSS has been validated in a previous cohort that included Australian medical students [8]. The tool has been used to examine knowledge gaps and inform education resources that improve suicide literacy for undergraduate medical students [8]. In a review of 25 studies that have used the LOSS across multiple countries, and with different cohorts of participants, the average proportion of items correctly answered was 63% (range 36.9–84.2%).

The Suicide of Stigma Scale (SOSS) is a tool that includes three subscales [3]. Participants are asked to respond to the statement: “using the scale below, please rate how much you agree with the descriptions of people who take their own lives (suicide). In general, people who suicide are...” [3]. What follows are 58 descriptive words in the domains of stigma items (*n* = 31), isolation and depression items (*n* = 16), and glorification and normalization items (*n* = 11). Participants have the option to respond with one of five Likert-scale statements (1 = strongly disagree, 2 = disagree, 3 = neutral, 4 = agree, 5 = strongly agree). The tool as a whole has previously demonstrated strong internal consistency of 0.75 to 0.93 with different cohorts of participants, including Australian medical students [3]. Internal consistency of subscales has also been strong (stigma subscale 0.95–0.96; isolation and depression subscale 0.88–0.93; glorification and normalization subscale 0.86–0.88) [3, 24].

The General Help-Seeking Behaviour Scale developed by Wilson et al. [32] and also adopted by Chan et al. [10] was included. Participants respond to the question—“If you were experiencing suicidal thoughts, how likely is it that you would seek help from the following people?” and are instructed to rate the included people on a four-point scale where 1 = highly unlikely; 2 = unlikely; 3 = likely; and 4 = highly likely [32].

The confidence questions from the Attitudes and Confidence in the Integration of Psychiatry Scale were included [17]. This questionnaire has a Cronbach’s alpha of 0.90 and was developed for administration to third and fourth-year medical students [17]. Participants rate each item on a seven-point Likert scale (1 = not capable; 7 = extremely capable). The authors recognize the limitations of these questions as self-perceptions of competence, and not actual skill, however, in this study, we wished to understand participants’ baseline and changes in self-perceived confidence in relation to their psychiatry rotation following the workshop so as to inform future iterations.

Finally, in the post-workshop questionnaires, participants were additionally asked to complete the Satisfaction with Simulation Experience Scale (SSES) [22]. The SSES is a tool containing 18 items in the domains of Clinical Learning, Clinical Reasoning, and Debrief and Reflection. Participants rated their level of agreement with statements on a 5-point Likert scale ranging from strongly agree (5) to strongly disagree (1).

### Methodological considerations

This study was a collaboration between the Adelaide Medical School (AMS) (SC, NM) and AHS (ED, AM), with support from the Adelaide School of Psychology (ACH). As educators and researchers, we hoped that measures of stigma would be reduced and suicide literacy would improve after the simulation workshop. However, we also acknowledge that a single learning event is unlikely to, by itself, significantly shape attitudes, confidence, and clinical capability: students exist within their own social contexts and are exposed to a broader curriculum and the clinical environment. We anticipated the workshop would provide a valuable opportunity for students to reflect on their responses to potentially distressing situations and provide an opportunity to reduce anxiety before entering clinical practice as a student.

Existing tools were adopted to reduce known and potential researcher biases. These tools have all been validated in previous studies in similar settings (pre-registration health professions in Australian universities). Given the similarities between our cohort of participants, and the populations of people that were used in the validation of these tools, we make the assumption that the use of these tools in this study would be sufficient to address the research questions and would produce similar validity. The questionnaires used in this study are self-reported, and therefore, findings will potentially include participant biases towards socially expected answers. To reduce this risk, data were de-identified by the lead author (ED) prior to data analysis and those with direct teaching connections with the cohort (SC and NM) did not have access to identifiable data. Participants were notified of these processes prior to completing surveys.

### Resource and cost considerations

Adelaide Health Simulation is an established service that has existing clinical and technical expertise, infrastructure, and equipment to facilitate small and larger group learning using a wide variety of simulation modalities and technologies. For this project, we used existing VR headsets, an Igloo 360° immersive cylinder, and our on-campus simulation facilities. The videos used for station 1 had been produced by the AHS team for a different project and were integrated into this project as relevant and supportive teaching materials. Costs directly related to this project include people’s time to design and deliver the workshops and time for editing the video for LELAN for the 360° Igloo immersive cylinder. Approximated, itemized costs are provided in Supplementary file 3. These are separated into the costs for infrastructure and equipment that were available prior to the conception of these workshops and direct costs for the design and delivery of workshops. Consultant psychiatrists provided in-kind support as facilitators of the workshops.

### Study size

As a single-center study, the study size was dictated by the number of students enrolled in the program and who attended the simulation workshops. A convenience sample was sought.

### Data analysis

Demographic data are summarized and presented in Table 2. The percentages of participants with “correct,” “incorrect,” and “unsure” answers for the LOSS items, both pre- and post-workshop were calculated for each item. The mean and standard deviations for “correct” answers within each of the four scale domains (causes and nature/triggers, risk factors, signs and symptoms, and treatment and prevention) were calculated. Percentage agreement, means, and standard deviations were also calculated for the pre- and post-workshop responses to items on the SOSS. Paired *t* tests were used to compare pre- and post-workshop findings from the SOSS for those participants who completed both the pre- and post-workshop. Welch’s *t* test was used to compare pre- and post-workshop findings from the SOSS regardless of whether they completed the survey at both time points (Supplementary file 2). Likert data from participants’ self-assessed level of capability when undertaking different clinical assessments in the psychiatric context (for example, “screening for depression”) are presented in a bar graph, as are findings from the help-seeking scale. Finally, means and standard deviations are presented for the SSES. Microsoft Excel was used for all data analysis and chart development.

**Table 2:** Demographic information from questionnaire participants

	**Pre-workshop questionnaire** **(total number 116)**	**Post-workshop questionnaire** **(total number 60)**
Age (mean; range)	22.05; 19–31^a^	21.95; 19–31
Gender	***n* (%)**	***n* (%)**
*Female*	65 (56.0)	35 (58.3)
*Male*	50 (43.1)	25 (41.7)
*Non-binary*	1 (0.9)	0 (0)
Country of birth	***n* (%)**	***n* (%)**
*Australia*	59 (50.9)	32 (53.3)
*India*	15 (12.9)	5 (8.3)
*Singapore*	12 (10.3)	7 (11.7)
*Malaysia*	9 (7.8)	5 (8.3)
*Republic of Korea*	5 (4.3)	3 (5.0)
*England*	2 (1.7)	0 (0)
*Sri Lanka*	2 (1.7)	0 (0)
*Other*^b,c^	7 (6.0)	0 (0)

^a^Missing one entry

^b^Pre-workshop questionnaire, 1 participant each from Bangladesh, China, Iran, Kuwait, New Zealand, Thailand, and the USA

^c^Post-workshop questionnaire, 1 participant each from Bangladesh, China, Iran, New Zealand, Thailand, and the USA

### Project reporting

Cheng et al.’s [11] guidelines for reporting healthcare simulation research guided the reporting of this study.

### Ethics

This study was approved by the UoA Lower Risk Ethics Committee (HREC-2022–182). Participation was voluntary. Participants who completed both pre- and post-workshop surveys and who provided a student ID number went into a draw to win one of two $50 electronic eftpos vouchers in each rotation group.

## Results

### Participants

Data were collected between March and December 2023 from a total of 118 participants (68.60% of the total cohort). Pre-workshop questionnaires were completed by 116 participants, and post-workshop questionnaires were completed by 60 participants. Participants’ demographic information is provided in Table 2.

### Suicide literacy

As a cohort, correct answers on the LOSS increased from a mean percentage of 65.8% in the pre-workshop survey, to 70.1% in the post-workshop survey (Table 3), indicating small gains in suicide literacy. In both pre- and post-surveys, correct responses in the treatment and prevention domains were by far the highest (92.9% and 95.0% correct responses, respectively). Participants were least knowledgeable about the signs and symptoms of suicidality (38.0% and 44.2% correct responses, respectively).

**Table 3:** Pre- and post-workshop LOSS findings

Theme	No. of items	Mean % correct pre-workshop (116 participants)	SD	Mean % correct post-workshop (60 participants)	SD
Causes and nature/triggers	10	74.0	24.6	79.3	24.7
Risk factors	7	62.5	23.6	65.0	18.8
Signs and symptoms^a^	6	38.0	22.1	44.2	22.0
Treatment and prevention	4	92.9	1.93	95.0	2.7
**Total**	**27**	**65.8**		**70.1**	

^b^Included in Chan et al. [10] but notCalear et al. [8]

When viewing data for individual items on the LOSS (Supplementary file 2, Supplementary Table 3), participants more frequently indicated they were “unsure” than selecting an incorrect answer in almost all instances. In both pre- and post-workshop surveys, the items “a person who suicides is mentally ill” and “suicide rarely happens without warning,” incorrect answers were selected more frequently than correct answers. Suicide literacy was low for some dynamic risk factors such as “anxiety and agitation” and “ambivalence and fluctuation in intent.”

### Suicide stigma

Fifty-nine participants completed both the pre- and post-SOSS. Table 4 outlines the percentage agreement and mean responses for each item of the stigma subscale of the SOSS for those participants who completed both surveys. Findings from the isolation and depression and the glorification and normalization subscales can be found in Supplementary file 2 (Supplementary file 2, Tables 2), as can findings from the entire participating cohort for all three subscales (Supplementary file 2, Tables 3 and 4).

**Table 4:** Pre- and post-workshop SOSS findings (stigma items)

Item	Pre-workshop Questionnaire		Post-workshop Questionnaire		*T* test	
	**% Agreement**	**Mean (SD)**	**% Agreement**	**Mean (SD)**	***t***	***p***
**Stigma items**						
**Reckless**	10.43	2.05 (1.66)	5.08	1.68 (0.93)	2.75	0.00*
**Selfish**	7.76	1.86 (1.52)	5.08	1.58 (0.82)	2.30	0.01*
**Punishing others**	6.90	1.98 (1.41)	3.39	1.72 (0.88)	1.93	0.03*
Hurtful	6.90	1.84 (1.46)	10.17	1.88 (1.07)	-0.25	0.40
Unjustifiable	5.17	1.74 (1.27)	3.39	1.58 (0.68)	1.32	0.09
Irresponsible^a^	4.35	1.79 (1.04)	6.78	1.61 (0.86)	1.60	0.06
**Unnatural**	3.45	1.65 (0.95)	0.00	1.39 (0.42)	2.22	0.02*
Immoral	3.45	1.60 (0.82)	1.69	1.47 (0.54)	1.41	0.08
Unfair^a^	3.48	1.59 (0.94)	5.08	1.63 (0.86)	-0.31	0.38
Cruel	3.45	1.56 (0.75)	1.69	1.47 (0.61)	0.87	0.19
Senseless	3.45	1.49 (0.72)	3.39	1.47 (0.65)	0.18	0.43
**Cowardly**	2.59	1.51 (0.68)	0.00	1.35 (0.34)	1.76	0.04*
Ignorant	1.72	1.53 (0.62)	0.00	1.42 (0.46)	1.18	0.12
Shallow	1.72	1.47 (0.56)	0.00	1.44 (0.46)	0.42	0.34
A burden	1.72	1.40 (0.57)	0.00	1.35 (0.34)	0.60	0.28
An embarrassment	0.86	1.35 (0.45)	0.00	1.25 (0.26)	1.35	0.09
Strange	0.86	1.47 (0.65)	0.00	1.44 (0.46)	0.47	0.32
Attention-seeking	0.00	1.53 (0.58)	0.00	1.51 (0.54)	0.15	0.44
**Stupid**	0.00	1.47 (0.47)	0.00	1.33 (0.33)	1.74	0.04*
Weak^a^	0.00	1.46 (0.40)	1.69	1.54 (0.54)	-0.85	0.20
Vengeful	0.00	1.40 (0.42)	0.00	1.39 (0.38)	0.24	0.41
Arrogant^a^	0.00	1.38 (0.42)	0.00	1.36 (0.42)	0.21	0.42
Shameful	0.00	1.35 (0.30)	0.00	1.28 (0.31)	1.07	0.14
Lazy	0.00	1.35 (0.34)	0.00	1.44 (0.46)	-1.04	0.15
Unforgivable	0.00	1.33 (0.33)	0.00	1.30 (0.36)	0.57	0.28
Violent	0.00	1.32 (0.36)	0.00	1.44 (0.43)	-1.47	0.07
Pathetic	0.00	1.32 (0.33)	0.00	1.32 (0.33)	0.00	0.50
Failures	0.00	1.32 (0.26)	0.00	1.26 (0.27)	0.90	0.19
Evil	0.00	1.30 (0.36)	0.00	1.23 (0.25)	0.89	0.19
Barbaric^a^	0.00	1.30 (0.36)	0.00	1.32 (0.40)	-0.26	0.40
Useless	0.00	1.26 (0.23)	0.00	1.26 (0.30)	0	0.50

5-point Likert-scale: 1 = strongly disagree; 2 = disagree; 3 = neutral; 4 = agree; 5 = strongly agree

^a^Sample size of 58

^*^Significant *p* (< 0.05)

Low levels of agreement with the stigma items of the SOSS were identified in both pre- and post-workshop surveys. Pre-workshop, only four items exceeded an overall agreement of 10% (Reckless, Hurtful, Punishing others, Selfish) (Supplementary File 2). In the paired *t* test findings, scores with statistically significant findings included the items “stupid,” “cowardly,” “unnatural,” “selfish,” “reckless,” and “punishing others” (Table 4).

### Self-perceived capability of performing clinical assessments

**Fig. 1:**
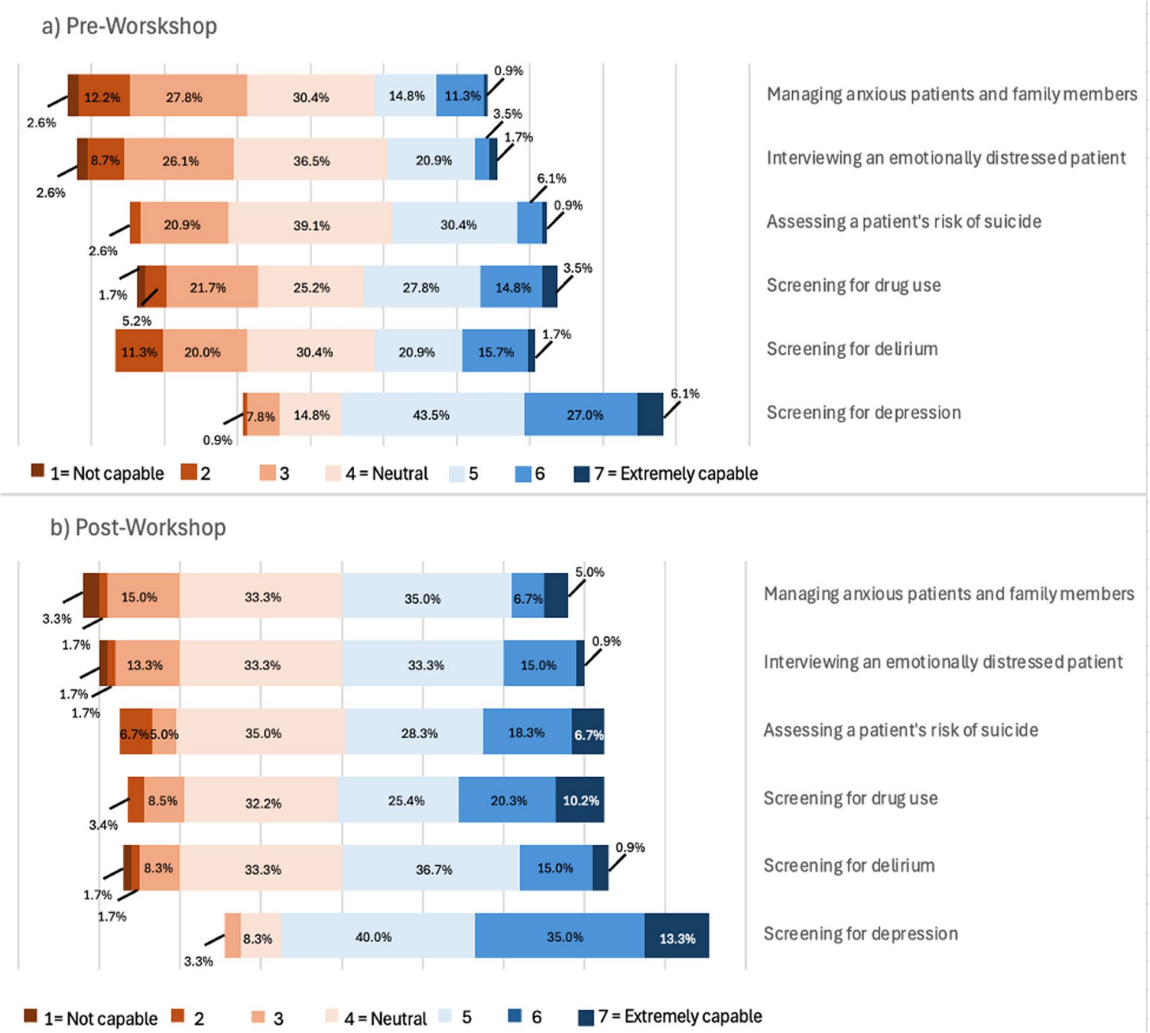
Self-perceived level of capability when undertaking different clinical assessments

Participants’ self-perceived level of capability to assess and manage patients in a mental health setting is presented in Fig. 1. Findings from the Likert-scale responses are presented as percentages for pre- (*n* = 118) and post-(*n* = 60) workshop participants. Notably, students felt most capable of screening for depression and least capable of managing anxious patients and family members at both time points.

### Help-seeking behavior

**Fig. 2:**
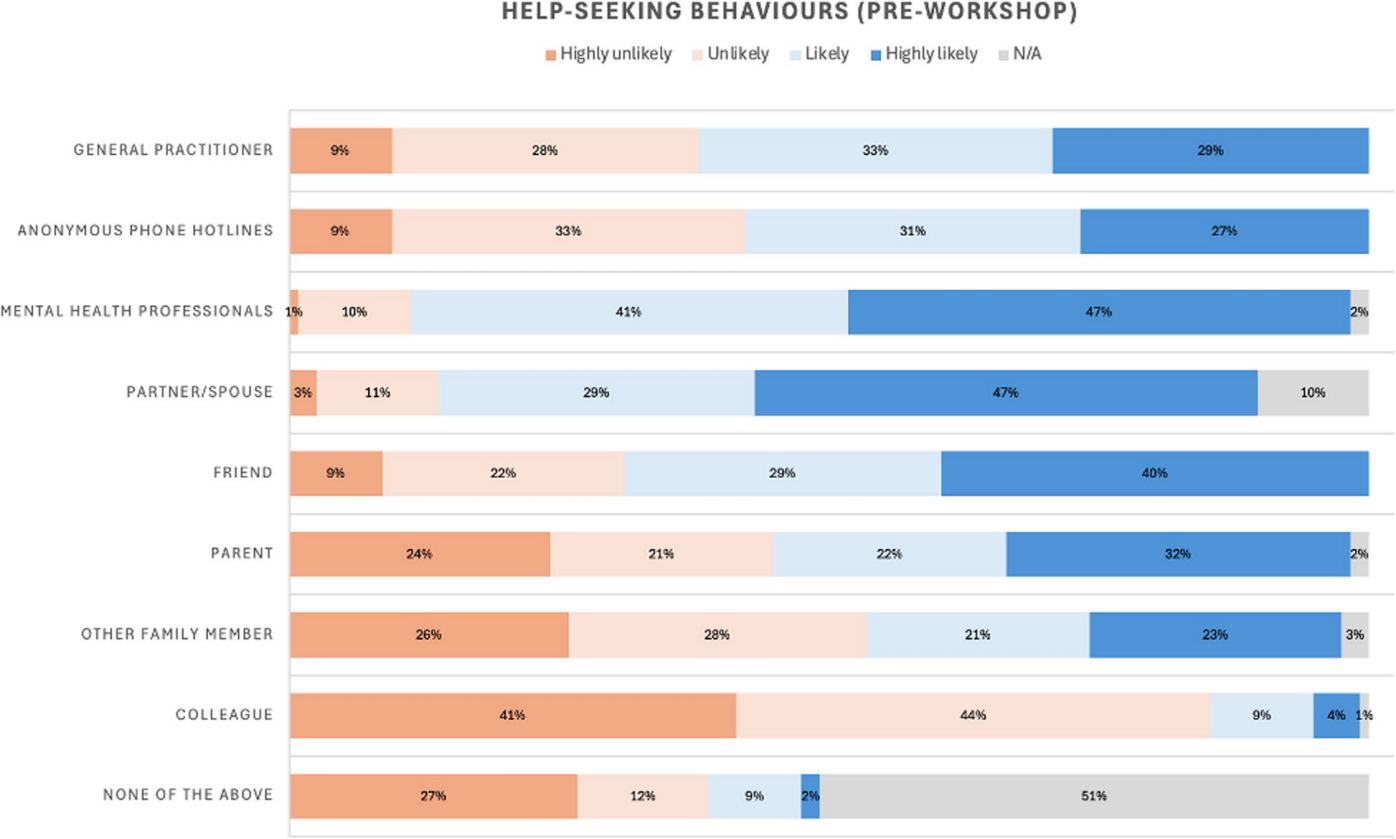
Help-seeking behaviors

Percentages for both pre- and post-help-seeking questions were almost identical, and so, the pre-workshop data with a larger percentage of participation (116; 67.4%) are presented (Fig. 2). The majority of participants indicated they were either likely or highly likely to seek help from a mental health professional and their partner or spouse in the event of experiencing mental distress (88% and 76%, respectively). Over half of the cohort indicated they would seek help from a general practitioner (62%), an anonymous phone hotline (58%), or a parent (54%). Over 80% indicated they would likely not confide in a colleague. Around one-tenth (11%) of participants indicated they were unlikely or highly unlikely to contact any of the listed potential “helpers.”

### Satisfaction with simulation experience

In the post-workshop questionnaire, participants were asked to complete the Satisfaction with Simulation Experience Scale in addition to the other scales (Table 5). Students were generally satisfied with the workshop. The five highest-rated items were from the debrief and reflection domain, likely indicating strong satisfaction with the opportunity to engage in discussion and debriefs with consultant psychiatrists. The lowest-rated items largely related to the clinical reasoning domain. In a workshop where only a few students had opportunities to be actively involved in the SP interaction, and the other stations were largely observational, this is somewhat unsurprising.

**Table 5:** Satisfaction with simulation experience scale–post-workshop

Statement number	Domain	Statement	Mean (SD)
13	**DR**	The debriefing provided an opportunity to ask questions	4.48 (0.54)
18	**DR**	The facilitator made me feel comfortable and at ease during the debriefing	4.45 (0.72)
11	**DR**	The facilitator summarised important issues during the debriefing	4.42 (0.62)
9	**CR**	This was a valuable learning experience	4.33 (0.60)
15	**DR**	Reflecting on and discussing the simulation enhanced my learning	4.32 (0.71)
16	DR	The facilitator’s questions helped me to learn	4.27 (0.69)
1	CL	The simulation caused me to reflect on my clinical ability	4.22 (0.52)
12	DR	I had the opportunity to reflect on and discuss my performance during the debriefing	4.15 (0.76)
10	DR	The facilitator provided constructive criticism during the debriefing	4.15 (0.76)
14	DR	The facilitator provided feedback that helped me to develop my clinical reasoning skills	4.10 (0.71)
4	CL	The simulation helped me to recognize my clinical strengths and weaknesses	4.00 (0.78)
17	DR	I received feedback during the debriefing that helped me to learn	3.98 (0.89)
3	CL	The simulation helped me to apply what I have learned	3.97 (0.76)
5	CR	The simulation developed my clinical reasoning skills	3.95 (0.70)
6	CR	The simulation developed my clinical decision-making ability	3.87 (0.79)
2	CL	The simulation tested my clinical ability	3.78 (0.91)
8	CR	The simulation helped me to recognize patient deterioration early	3.62 (0.96)
7	CR	The simulation enabled me to demonstrate my clinical reasoning skills	3.55 (1.05)

5-point Likert-scale: 1 = strongly disagree; 2 = disagree; 3 = unsure; 4 = agree; 5 = strongly agree

*CL* clinical learning domain, *CR* clinical reasoning domain, *DR* debrief and reflection domain, *Q* question number

## Discussion

Medical student preparation for entering a psychiatry rotation and associated clinical placement is important. Recovery-oriented models of care, trauma-informed, patient-centered care, and the implementation of evidence-based practice in mental health units cannot occur if students and practitioners are influenced by stigma and are unaware of the potential system impact on individuals experiencing mental distress [12, 21]. Student readiness for practice in this environment can be considered from multiple perspectives, including what they know about mental illness and the field of psychiatry, what they can do as medical students, and what they value when working in mental health contexts [5].

In this study, fourth-year medical students participated in surveys before and after attending a newly developed hybrid simulation workshop. This workshop was designed to highlight the impact that healthcare professionals can have on patient trajectories through health systems, explain the roles and responsibilities of different members of the healthcare team in assessing and managing patients exhibiting behaviors of concern, provide opportunities to hear from people with lived experiences of suicidal thoughts and self-harm behaviors, and provide an opportunity to practice assessment and communication skills with a simulated patient.

Existing surveys were administered to evaluate suicide literacy, suicide stigma, capability for assessing and managing people who have presented with a mental illness, self-help-seeking behaviors, and satisfaction with the simulation workshop. Findings suggest that this novel, multifaceted, and immersive intervention produced some small but statistically significant improvements in learners’ understanding of suicide and self-harm. We noted an increase in suicide literacy, with the percentage of correct answers on the LOSS rising from 65.8 to 70.1% post-workshop. For both time points, the percentage of correct answers was above the average (63%) for university students in previous studies that have used the LOSS [8].

Post-intervention, there were small but significant decreases in the identification of patients who self-harm as being “selfish” or “stupid” and a small significant increase in the identification of suicide as “rational” post-intervention. It was identified that suicide literacy was low for some dynamic risk factors such as “anxiety and agitation” and “ambivalence and fluctuation in intent.” While these issues are addressed in lectures and tutorials in the orientation week for the course in the week prior to the workshop, it seems that more emphasis needs to be placed on dynamic risk factors in clinical contexts.

Students reported feeling very capable of screening for depression, and there were improvements in self-assessed capability to assess and manage people experiencing a mental illness. This finding is somewhat at odds with findings from the LOSS, which identified knowledge gaps relating to risks, particularly dynamic risks for suicide. The self-reported findings of high confidence for this particular item may reflect students’ affinity for using structured assessment forms with patients and/or may reflect a gap in their understanding of their actual clinical capability.

Students were particularly satisfied with the opportunity to engage with consultant psychiatrists. As curricula have transitioned to more digital platform-based teaching following the COVID-19 pandemic, our results highlight in contrast the value students place on expert consultant teaching, complementing findings from other recent studies and commentaries [15, 31].

Our intervention did not intend to, nor did it substantially change students’ likely help-seeking-behaviors. What the study highlights is a critical need for further professional development and support regarding the importance of mental health self-care. While the majority of participants suggest they would seek specialist support, over 40% would not confide in a general practitioner, use an anonymous hotline, or even confide in a parent, with 11% suggesting they would not seek any external help and over 80% indicated they would likely not confide in a colleague. These results further highlight that medical students remain at risk of concealing serious mental health issues likely due to concern of ramifications for career progression [4]; [10]).

Simulation can facilitate targeted teaching around relatively rare events such as suicidality and optimize the impact of experienced clinician educators. There are opportunities to increase this type of teaching across more domains in the psychiatry rotation (not only self-harm and suicidality). The workshop itself is brief and has only one intervention point. Consideration of the medical curriculum as a whole is important. We recognize the importance of introducing concepts and content early in the medical degree, so that by the time students arrive at their placements, mental health literacy is higher, and stigma is reduced as much as possible.

Different simulation modalities and the various immersive technologies can be excellent vehicles for facilitating learner readiness for contexts, like mental health wards and services, where there are known stigmas, and misconceptions [1, 9, 13, 19, 28]. In this workshop, the lived experiences of people who have experienced mental illness were given voice and likely contributed to the small reductions in stigma seen in the study, increased mental health literacy, and patient perspectives about the context medical students would be entering on placement. The impacts of immersive exposure may help frame attitudes as clinical expertise develops, and the impact may be on longer term outcomes. While difficult to measure, we hope that the final impact is a reduction of stigma, an improved trauma-focused understanding of care, and ultimately less restrictive management appropriate to the level of risk. Alternatively, our short-term gains may be short-lived and driven by external or more stable internal factors, hence harder to shift over time.

There are opportunities to extend this workshop to include students from other health professions (for example nursing, psychology, pharmacy, allied health), both for developing their attitudes towards suicidality and self-harm, and to provide opportunities to learn with, from and about other student professionals who will become future colleagues in multidisciplinary teams. There are also opportunities to examine and compare other student cohorts. This study was conducted in a medical school that has a high percentage of school-leavers. Comparisons with student cohorts from post-graduate medical programs must be considered in light of the younger age of our participants, and perhaps, less life experience.

### Limitations

The scales used in this study are all self-report and are likely to suffer biases towards socially desirable responses [20]. The time frame of pre- and post-test was short between 4 and 14 days to allow immediate changes in perspective to be analysed—long-term changes not measured, nor likely to be adequately captured when considering this event in the context of other learning opportunities. While our intervention was novel, immersive, and reinforced patient-centered and trauma-informed care, we only saw limited gains in scales assessing suicide literacy. Assessments designed to better analyze skills in trauma-informed care may be better able to highlight the benefits of this intervention—for example, the Attitudes Related to Trauma-Informed Care Scale (ARTIC) which is a self-report instrument for staff in human service, health, and educational settings [2]. The workshop was required as part of the curriculum, but involvement in the study was voluntary—and while this is entirely appropriate, it means that the data may not represent all views across the cohort. The loss to follow-up is one aspect of this.

## Conclusion

Medical students will encounter people who experience mental illness, thoughts of suicide, and self-harm behaviors throughout their careers. Beliefs and values about mental illness will shape how these developing health professionals will engage with patients experiencing mental distress. Evidence suggests that students should have early access to resources and experts, including those with lived experience, to shape their understanding of this field and the experiences of patients, to reduce stigma, and to improve attitudes towards this field of medicine. In this study, a novel approach was taken to integrate perspectives of people with lived experiences of mental illness, suicide, and self-harm behaviors. Immersive simulation technologies, including virtual reality and simulated patients, were included in a half-day workshop prior to student placement in mental health units to facilitate student understanding that their attitudes and actions as medical officers will impact patient trajectories and outcomes. The workshop provided opportunities to ask questions of practicing psychiatrists and become more aware of context. Findings suggest that the workshop reduced some areas of stigma, improved suicide literacy, and was valued by students.

## Supplementary Information

Supplemenatary Material 1. Tutor Session Plan–AHS.

Supplemenatry Material 2. Table S1: Literacy of Suicide Scale (LOSS)–all items. Table S2: SOSS–isolation/depression and glorification/normalization subscales for paired T-test. Table S3: SOSS–all participants stigma subscale. Table 4: SOSS–isolation/depression and glorification/normalisation subscales–all participants.

Supplementary Material 3. Indicative costs for fourth year MBBS student workshops.

## Data Availability

The data that support the findings of this study are available from the corresponding author but restrictions apply to the availability of these data and so are not publicly available. Data are however available from the authors upon reasonable request and with permission of the corresponding author.

## Abbreviations

AHS: Adelaide Health Simulation
AMS: Adelaide Medical School
LELAN: Lived Experience Local Advocacy Network
LOSS: Literacy of Suicide Scale
SSES: Satisfaction with Simulation Experience Scale
SOSS: Stigma of Suicide Scale
SP: Simulated patient
UoA: University of Adelaide
VR: Virtual reality
