# Supplementary material for: Hybrid simulation and immersive, lived-experience perspectives to shape medical student attitudes towards patients experiencing emotional distress, suicidality, and self-harm
Source: Adv Simul (Lond). 2025 Mar 24;10:13. doi: 10.1186/s41077-025-00336-4 (PMC11931875; doi:10.1186/s41077-025-00336-4)
Supplement: Supplementary file 1 — Supplemenatary Material 1. Tutor Session Plan–AHS. [file 41077_2025_336_MOESM1_ESM.pdf]

THE UNIVERSITY of ADELAIDE

Adelaide Health Simulation

Tutor Session Plan

SESSION INFORMATION	
Program:	MBBS 4 2023
Rotation:	Psychiatry
Date(s) &<br>Time(s):<br>Location (s):	Date<br>Time – 1200 - 1700<br>Location – Helen Mayo South (Level 1)

INSTRUCTOR DETAILS	
Simulation Coordinator:	Libby Kentish
Course Coordinator:	Scott Clark / Natalie Mills
Additional Academics attending:	1 Academic (3 in total)
Simulated Patients required:	1 SP

LEARNING OBJECTIVES

- Demonstrate confidence and capacity to engage with people with self-harm behaviours, suicidal idealation or who have attempterd suicide. (SP scenario)
- Demonstrate a structured assessment of a patient presenting with signs and symptoms of a mental illness and present an accurate handover and risk assessment to a colleague. (SP scenario)
- Describe the roles and responsibilities of different members of the healthcare team in managing patients exhibiting behaviours of concern. (VR station)
- Describe in detail the impact that healthcare professionals have on patient progress through health systems. (LELAN video & VR station)

LESSON PLANS

30 students will meet in debrief A & B for introduction to the session, psychological safety discussed (10 – 15 mins)

They will be split into 3 groups, will rotate through 3 stations

Station 1. 360 Immersion room video – LELAN 40 min video & 30 min debrief

(70 mins)

Rotation – 5 mins

Station 2. VR – Self harm video x 2 versions – 10 min orientation by tech. 10 min plus 20 min debrief for each video (70 mins)

Break – 10 min bio breakl

Station 3. Scenario with SP - 'ED cubical' setting followed by debrief

(70 mins)

Students will meet in Debrief A & B for conclusion of the session

(10 – 15 mins)

Staffing allocation:

Station 1 – 1 x tutor, Station 2 – 1 x tutor, Station 3 – 1 x tutor & 1 x SP

PREBRIEF

Thank you for attending this workshop.

By the end of today's workshop and your placement, we would like you to be able to achieve the following:

- Demonstrate confidence and capacity to engage with people with self-harm behaviours, suicidal ideation or who have attempterd suicide.
- Demonstrate a structured assessment of a patient presenting with signs and symptoms of a mental illness and present an accurate handover and risk assessment to a colleague.
- Describe the roles and responsibilities of different members of the healthcare team in managing patients exhibiting behaviours of concern.
- Describe in detail the impact that healthcare professionals have on patient progress through health systems.

Station 1 will be in the immersion room, where you will be watching a video that has been created by the SA Lived Experience Leadership and Advocacy Network (LELAN). It features a number of people who have experienced mental distress or supported people who have experienced mental distress. It describes, in detail, the interactions that interviewees have had with health professionals, and the impact these interactions have had on mental health and recovery. *There are some words that are muted by the makers of the film – the times where the audio is muted are when interviewees talk about specific locations or people.

Station 2 involves watching two virtual reality videos. These videos do contain images of a simulated patient who is experiencing mental distress, and does self-harm by simulating cutting herself during the video. There is a small amount of blood present at this time.

The purpose of presenting this content to you is for you to have an opportunity to watch a patient's journey before they arrive in a hospital, and to critique the care and interactions that the patient has with health professionals. After each video, you will have the opportunity to discuss what you have seen, the behaviours of concern and the healthcare management.

In **session 3**, two students from your group will interview the patient from the VR videos for 15 minutes. You will be assigned to interview 'Rachel' using the 'Structured approach to clinical problems in psychiatry', and establish any immediate considerations (safety and medical emergencies), and history (i.e. steps 2 and 3 of the structured approach). Observers will be tasked with taking notes from the interview, and constructing a mental state assessment (step 5). All students will be involved in a debrief that follows the SP interview.

<u>**Information for Students – to be provided by the tutor in the room**</u>

If at any time you feel uncomfortable with the content of this workshop, need a break, or would like to step out of one of the stations, please know that we are here to support you. You can approach any one of the staff here today,

with your concerns, and we will support you to either continue to attend, to have a break from, or to discontinue your involvement in the workshop.

INSTRUCTOR SESSION GUIDES

Station 1:
Students will watch the LELAN video in the immersion room. This will take about 40 mins. There will be 30 mins for a discussion. The students should be asked for their thoughts after watching the video.
Care not Treatment | LELAN

The following points should be covered in the discussion:

- 'language' and risk – risk vs safety language
- Establishing trust in those who have been 'let down' by the system

Station 2:

Students will watch the two versions of the self harm video of the VR headset.

Each students will be in a room (room 1 – 5, 6, 9, 10 Debrief a & b). They will watch video 1 (10 mins) followed by 20 min debrief in debrief A. They will then watch video 2 (10 mins) followed by 20 min debrief in debrief A.

- Open discussion with initial reactions to the video. Following the first scenario, explore why Rachel ended up in seclusion
- The discussion should focus on the behaviours of concern and the healthcare management in both videos.
- Suggestions:

- What was Rachel feeling during the self-harm event?
- What happened on arrival on the paramedics?
- Was rapport established?
- What choices was Rachel offered?
- Could this have made a difference?
- How does the pre-hospital treatment affect hospital treatment?
- Where was she managed on arrival to ED?
- Was this appropriate?
- How did Rachel react to this?

Station 3: Structured assessment for mental health scenario. 15 min interaction with patient followed by a handover to the doctor of assessment. There will be a debrief involving all students and the SP.

Students are in a ED cubicle interviewing Rachel immediately following the 2nd VR example.

- 2 students to undertake the assessment for 15 minutes (to follow steps 2 & 3 - assessing for safety and gathering information)
- Remainder of viewers are to use the "Structured approach to clinical problems in psychiatry" model to evaluate the any immediate considerations (safety, medical emergencies) and to gather a history of the current presentation. Student viewers to put together a mantal state exam
- As a group, students can then discuss management options based on the formulation: "Immediate", "Short / Intermediate", and "Long term".

Tutor Session Plan

SETUP INFORMATION

FLOOR PLAN

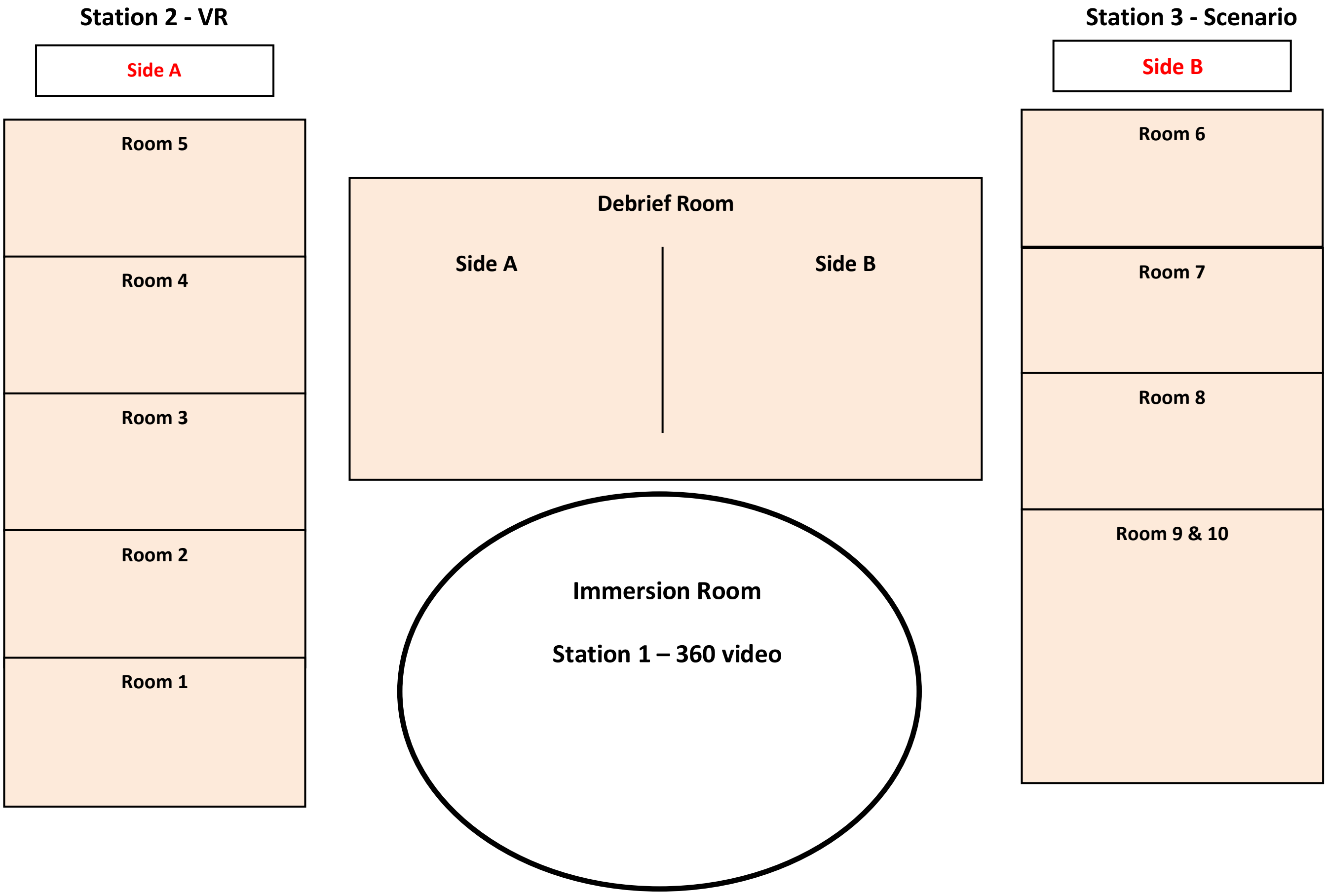

NOTES

Date:	Dec 2022/Jan 2023
Author:	L Kentish / S Jolly
Reviewed:	S Clark, A Montagu, E Davies

REFERENCES:
