# Supplementary material for: Hybrid simulation and immersive, lived-experience perspectives to shape medical student attitudes towards patients experiencing emotional distress, suicidality, and self-harm
Source: Adv Simul (Lond). 2025 Mar 24;10:13. doi: 10.1186/s41077-025-00336-4 (PMC11931875; doi:10.1186/s41077-025-00336-4)
Supplement: Supplementary file 2 — Supplemenatry Material 2. Table S1: Literacy of Suicide Scale (LOSS)–all items. Table S2: SOSS–isolation/depression and glorification/normalization subscales for paired T-test. Table S3: SOSS–all participants stigma subscale. Table 4: SOSS–isolation/depression and glorification/normalisation subscales–all participants. [file 41077_2025_336_MOESM2_ESM.pdf]

Supplementary File 2: Additional results

Supplementary Table 1: Literacy of Suicide Scale (LOSS) – all items

#	Item	Domain	Pre-workshop			Post-workshop (%)		
			Correct n (%)	Incorrect n (%)	Unsure n (%)	Correct n (%)	Incorrect n (%)	Unsure n (%)
2	Those who attempt suicide do so only to manipulate others and attract attention to themselves (F)	C/N	114 (98.3)	1 (0.9)	1 (0.9)	59 (98.3)	0 (0.0)	1 (1.7)
24	Nothing can be done to stop people from making the attempt once they have made up their minds to kill themselves (F)	T/P	109 (94.0)	0 (0)	6 (5.2)	57 (95.0)	0 (0.0)	3 (5.0)
25	Only experts can help people who want to suicide (F)	T/P	109 (94.0)	1 (0.9)	6 (5.2)	59 (98.3)	0 (0.0)	1 (1.7)
26	People who have thoughts about suicide should not tell others about it (F)	T/P	107 (92.2)	4 (3.4)	5 (4.3)	57 (95.0)	0 (0.0)	3 (5.0)
5	A suicidal person will always be suicidal and entertain thoughts of suicide (F)	C/N	106 (91.4)	3 (2.6)	7 (6.0)	56 (93.3)	2 (3.3)	2 (3.3)
6	Talking about suicide always increases the risk of suicide (F)	C/N	106 (91.4)	1 (0.9)	9 (7.8)	59 (98.3)	0 (0.0)	1 (1.7)
1	If you asked someone directly 'Do you feel like killing yourself?' it will likely lead that person to make a suicide attempt (F)	C/N	105 (90.5)	3 (2.6)	8 (6.9)	59 (98.3)	0 (0.0)	1 (1.7)
27	Seeing a psychiatrist or psychologist can help prevent someone from suicide (T)	T/P	104 (90.4)	3 (2.6)	8 (7.0)	55 (91.7)	4 (6.7)	1 (1.7)
13	A person who has made a past suicide attempt is more likely to attempt suicide again than someone who has never attempted (T)	RF	100 (86.2)	5 (4.3)	11 (9.5)	47 (78.3)	5 (8.3)	8 (13.3)
3	Very few people have thoughts about suicide (F)	C/N	96 (83.5)	1 (0.9)	18 (15.7)	53 (88.3)	0 (0.0)	1 (1.7)
12	People with relationship problems or financial problems have a higher risk of suicide (T)	RF	95 (82.6)	4 (3.5)	16 (13.9)	51 (85.0)	3 (5.0)	6 (10.0)
18	Not all people who attempt suicide plan their attempt in advance (T)	S/S	93 (80.2)	4 (3.4)	19 (16.4)	50 (83.3)	3 (5.0)	7 (11.7)
4	If assessed by a psychiatrist, everyone who suicides would be diagnosed as depressed (F)	C/N	92 (79.3)	3 (2.6)	21 (18.1)	57 (95.0)	1 (1.7)	2 (3.3)
7	Motives and causes of suicide are readily and easily established (F)	C/N	92 (79.3)	6 (5.2)	19 (15.5)	49 (81.7)	4 (6.7)	7 (11.7)
11	Most people who suicide are psychotic (F)	RF	87 (75.0)	4 (3.4)	25 (21.6)	47 (78.3)	1 (1.7)	12 (20.0)
16	There is a strong relationship between alcoholism and suicide (T)	RF	80 (69.0)	4 (3.4)	32 (27.6)	39 (65.0)	3 (5.0)	18 (30.0)
8	Media coverage of suicide will inevitably encourage other people to attempt suicide (F)	C/N	75 (65.2)	16 (13.9)	24 (20.9)	40 (66.7)	4 (6.7)	16 (26.7)
14	Men are more likely to suicide than women (T)	RF	71 (61.2)	22 (19.0)	23 (19.8)	39 (65.0)	8 (13.3)	13 (21.7)
15	People who are anxious or agitated have a higher risk of suicide (T)	RF	51 (44.0)	10 (8.6)	55 (47.4)	32 (53.3)	8 (13.3)	20 (33.3)
20	People who want to attempt suicide can change their mind quickly (T)	S/S	49 (42.6)	20 (17.4)	46 (40.0)	34 (56.7)	9 (15.0)	17 (28.3)
21	Most people who suicide don't make future plans (F)	S/S	37 (31.9)	25 (21.6)	54 (46.6)	18 (30.0)	14 (23.3)	28 (46.7)
9	Most people who attempt suicide fail to kill themselves (T)	C/N	36 (31.0)	15 (12.9)	65 (56.0)	18 (30.0)	11 (18.3)	31 (51.7)
10	A person who suicides is mentally ill (F)	C/N	35 (30.2)	54 (46.6)	27 (23.3)	17 (28.3)	26 (43.3)	26 (28.3)
19	People who talk about suicide rarely commit suicide (F)	S/S	34 (29.3)	15 (12.9)	67 (57.8)	23 (38.3)	6 (10.0)	31 (51.7)
22	Suicide rarely happens without warning (T)	S/S†	28 (24.1)	54 (45.7)	35 (30.2)	16 (26.7)	26 (43.3)	18 (30.0)
17	Most people who suicide are younger than 30 (F)	RF	23 (19.8)	30 (25.9)	63 (54.3)	18 (30.0)	16 (26.7)	26 (43.3)
23	A time of high suicide risk in depression is at the time when the person begins to improve (T)	S/S	23 (19.8)	22 (19.0)	71 (61.2)	18 (30.0)	12 (20.0)	30 (50.0)
	**AVERAGE**		**65.82%**	**10.48%**	**23.66%**	**70.13%**	**9.69%**	**20.19%**

† included in Chan et al (2014), but not Calear et al. (2022)

Abbreviations: C/N = causes and/or nature of suicidality; RF = risk factor; S/S = signs and symptoms; T/P = treatment and prevention

Supplementary Table 2: SOSS – Isolation/depression and Glorification/normalisation Subscales for Paired T-test

Paired t-test

n=59

	Pre-workshop Questionnaire		Post-workshop Questionnaire		T-test	
**Item**	**Agreement (%)**	**Mean (SD)**	**Agreement (%)**	**Mean (SD)**	***t***	***p***
**Isolation/Depression Items**						
Lonely†	31.30	3.71 (0.83)	67.80	3.82 (1.17)	-0.76	0.23
Isolated	33.62	3.86 (0.80)	69.49	3.93 (0.89)	-0.60	0.28
Lost	35.34	3.98 (0.87)	74.58	4.05 (0.77)	-0.57	0.28
Disconnected	32.76	3.70 (1.07)	66.10	3.82 (1.11)	-1.04	0.15
Miserable	34.48	3.84 (0.92)	59.32	3.72 (1.13)	0.94	0.17
Sad	37.07	4.00 (0.93)	77.97	4.04 (0.57)	-0.34	0.37
Trapped	40.87	4.18 (0.75)	79.66	4.23 (0.71)	-0.49	0.31
Depressed	39.66	4.12 (0.82)	71.19	4.04 (0.75)	0.87	0.19
In pain	45.69	4.47 (0.58)	88.14	4.44 (0.43)	0.38	0.35
Hurt	44.83	4.33 (0.58)	84.75	4.32 (0.72)	0.17	0.43
Alienated	32.76	3.88 (0.79)	69.49	4.04 (0.86)	-1.70	**0.05**
Unhappy	42.24	4.30 (0.68)	84.75	4.23 (0.43)	0.75	0.23
Withdrawn	28.45	3.74 (0.70)	61.02	4.23 (0.43)	0.75	0.23
Cut-off	28.45	3.70 (0.78)	59.32	3.80 (0.78)	-0.93	0.18
Broken	24.14	3.33 (1.40)	44.07	3.39 (1.53)	-0.44	0.33
Disturbed†	22.81	3.20 (1.40)	44.07	3.18 (1.50)	0.144	0.44
**Glorification/Normalisation Items**						
Strong	6.90	2.51 (1.04)	11.86	2.65 (0.98)	-1.07	0.14
Noble†	0.87	2.00 (0.73)	0.00	2.09 (0.70)	-0.66	0.26
Dedicated†	2.59	2.18 (0.80)	5.08	2.32 (0.84)	-1.05	0.15
Brave	6.03	2.39 (1.13)	8.47	2.42 (1.00)	-0.30	0.38
Realistic	0.86	2.11 (0.74)	3.39	2.30 (0.78)	-1.47	0.07
Powerful	0.00	2.00 (0.75)	3.39	2.22 (0.79)	-1.75	**0.04**
Rational	0.00	1.91 (0.58)	2.19	2.19 (0.67)	-2.25	**0.01**
Motivated	6.09	2.29 (1.04)	10.17	2.53 (0.79)	-0.28	0.39
Committed	7.76	2.72 (0.88)	16.95	2.84 (0.74)	-1.22	0.11
Fearless†	2.59	2.13 (0.88)	3.45	2.27 (0.71)	-1.27	0.10
Understandable‡	14.66	2.89 (1.14)	29.31	2.91 (1.16)	-0.16	0.44

5-point Likert-scale: 1=Strongly Disagree; 2=Disagree; 3=Neutral; 4=Agree; 5=Strongly Agree
† sample size of 58; ‡ sample size of 57
* significant p (<0.05)

Supplementary Table 3: SOSS – All participants Stigma Subscale

Welch's t-test

	Pre-workshop Questionnaire		Post-workshop Questionnaire		T-test	
**Item**	**Agreement (%) (n=116)**	**Mean (SD)**	**Agreement (%) (n=59)**	**Mean (SD)**	***t***	***p***
**Stigma Items**						
Reckless	**13.91**†	1.89 (1.15) ‡	5.08	1.66 (0.96)	1.372	0.086
Hurtful	**12.93**	1.82 (1.14)	10.17	1.85 (1.03)	-0.167	0.434
Punishing others	**12.07**	1.89 (1.11)	3.39	1.69 (0.93)	1.212	0.114
Selfish	**12.07**	1.87 (1.12)	5.08	1.56 (0.90)	1.996	0.024*
Irresponsible	8.70†	1.78 (1.02) ‡	6.78	1.61 (0.93)	1.123	0.131
Unjustifiable	8.62	1.65 (1.01)	3.39	1.56 (0.82)	0.617	0.269
Cruel	6.90	1.56 (0.91)	1.69	1.46 (0.77)	0.783	0.218
Immoral	6.90	1.56 (0.90)	1.69	1.46 (0.73)	0.782	0.218
Unfair	5.22†	1.63 (0.90)‡	5.08	1.61 (0.91)	0.106	0.456
Senseless	5.17	1.44 (0.79)	3.39	1.46 (0.79)	-0.141	0.444
Unnatural	5.17	1.60 (0.90)	0.00	1.41 (0.67)	0.162	0.053
Ignorant	4.31	1.51 (0.83)	0.00	1.41 (0.67)	0.874	0.192
A burden	3.45	1.38 (0.72)	0.00	1.34 (0.63)	0.402	0.344
Cowardly	3.45	1.47 (0.81)	0.00	1.34(0.58)	1.275	0.102
Shallow	2.59	1.42 (0.70)	0.00	1.42 (0.67)	-0.012	0.495
Stupid	2.59	1.53 (0.79)	0.00	1.32 (0.57)	1.958	0.026*
Weak	1.74†	1.45 (0.70)‡	1.69	1.51 (0.73)	-0.488	0.313
Pathetic	1.72	1.34 (0.63)	0.00	1.31 (0.56)	0.421	0.337
Strange	1.72	1.41 (0.72)	0.00	1.42 (0.67)	-0.090	0.464
Unforgivable	1.72	1.31 (0.62)	0.00	1.29 (0.59)	0.231	0.490
An embarrassment	0.86	1.28 (0.57)	0.00	1.24 (0.50)	0.560	0.288
Attention-seeking	0.86	1.53 (0.74)	0.00	1.49 (0.73)	0.367	0.357
Lazy	0.86	1.32 (0.58)	0.00	1.42 (0.67)	-1.015	0.156
Shameful	0.86	1.33 (0.63)	0.00	1.27 (0.55)	0.609	0.272
Violent	0.86	1.33 (0.63)	0.00	1.42 (0.65)	-0.936	0.176
Arrogant	0.00†	1.31 (0.57)‡	0.00	1.34 (0.63)	-0.265	0.396
Barbaric	0.00†	1.27 (0.54)‡	0.00	1.31 (0.62)	-0.373	0.355
Evil	0.00	1.27 (0.55)	0.00	1.22 (0.49)	0.571	0.284
Failures	0.00	1.28 (0.52)	0.00	1.25 (0.51)	0.367	0.357
Useless	0.00	1.24 (0.47)	0.00	1.25 (0.54)	-0.155	0.439
Vengeful	0.00	1.39 (0.63)	0.00	1.37 (0.61)	0.152	0.440

5-point Likert-scale: 1=Strongly Disagree; 2=Disagree; 3=Neutral; 4=Agree; 5=Strongly Agree
† sample size of 115; ‡sample size of 114; §1 missing entry; ¶2 missing entries; #sample size of 58
* significant p<0.05

Supplementary Table 4: SOSS – Isolation/depression and Glorification/normalisation Subscales – All participants

Item	Pre-workshop Questionnaire		Post-workshop Questionnaire		T-test	
	Agreement (%) (n=116)	Mean (SD)	Agreement (%) (n=59)	Mean (SD)	*t*	*p*
**Isolation/Depression Items**						
In pain	92.24	4.46 (0.84)	91.53	4.44 (0.65)	0.141	0.444
Unhappy	88.79	4.28 (0.85)	86.44	4.22 (0.67)	0.471	0.319
Hurt	87.93	4.27 (0.85)	88.14	4.32 (0.84)	-0.388	0.349
Trapped	80.87†	4.07 (1.02)‡	81.36	4.22 (0.85)	-1.030	0.152
Depressed	77.59	4.03 (0.99)	72.88	4.03 (0.87)	0.004	0.498
Sad	75.00	3.93 (1.03)	79.66	4.03 (0.76)	-0.746	0.228
Alienated	71.55	3.94 (0.92)	71.19	4.03 (0.84)	-0.638	0.262
Lost	71.55	3.85 (1.13)	76.27	4.05 (0.88)	-1.272	0.103
Isolated	70.69	3.79 (0.99)	72.88	3.95 (0.94)	-1.021	0.154
Disconnected	66.38	3.71 (1.08)	67.80	3.83 (1.05)	-0.728	0.234
Lonely	65.22†	3.60 (1.08)‡	67.80	3.76 (1.12)	-0.918	0.180
Miserable	63.79	3.60 (1.16)	61.02	3.73 (1.06)	-0.714	0.238
Withdrawn	60.34	3.74 (0.94)	64.41	3.83 (0.95)	-0.588	0.279
Cut-off	57.76	3.66 (0.99)	61.02	3.80 (0.89)	-0.902	0.184
Broken	51.72	3.35 (1.22)	45.76	3.39 (1.25)	-0.184	0.427
Disturbed	42.11‡	3.15 (1.19)¶	45.79	3.15 (1.23)	-0.018	0.493
**Glorification/Normalisation Items**						
Understandable	28.45†	2.89 (1.01)‡	29.31#	2.89 (1.06)§	-0.040	0.484
Committed	18.97	2.68 (0.99)	18.64	2.83 (0.89)	-1.001	0.158
Motivated	14.78	2.50 (1.03)	11.86	2.53 (0.92)	-0.138	0.445
Brave	12.07	2.37 (1.03)	10.17	2.42 (1.02)	-0.324	0.373
Strong	11.21	2.49 (0.96)	11.86	2.66 (0.98)	-1.091	0.139
Fearless	6.03	2.29 (0.96)	3.45#	2.26 (0.85)§	0.242	0.405
Dedicated	5.17	2.23 (0.92)	6.78	2.34 (0.94)	-0.756	0.226
Realistic	5.17	2.18 (0.93)	3.39	2.27 (0.89)	-0.625	0.266
Powerful	4.31	2.12 (0.92)	3.39	2.20 (0.89)	-0.575	0.283
Noble	2.61†	2.05 (0.88)‡	0.00	2.10 (0.84)	-0.361	0.359
Rational	0.86	1.94 (0.78)	1.69	2.17 (0.81)	-1.790	0.038*

5-point Likert-scale: 1=Strongly Disagree; 2=Disagree; 3=Neutral; 4=Agree; 5=Strongly Agree
† sample size of 115; ‡ sample size of 114; § 1 missing entry; ¶ 2 missing entries; # sample size of 58
* significant $p<0.05$
