# Supplementary material for: Hybrid simulation and immersive, lived-experience perspectives to shape medical student attitudes towards patients experiencing emotional distress, suicidality, and self-harm
Source: Adv Simul (Lond). 2025 Mar 24;10:13. doi: 10.1186/s41077-025-00336-4 (PMC11931875; doi:10.1186/s41077-025-00336-4)
Supplement: Supplementary file 3 — Supplementary Material 3. Indicative costs for fourth year MBBS student workshops. [file 41077_2025_336_MOESM3_ESM.pdf]

Supplementary File 3 – Indicative costs for Fourth Year MBBS Student Workshops

Existing infrastructure and resources*

Item description	Year of purchase	Cost per item**	# of items	Total cost
**Igloo 360° immersive cylinder**	2019	$283000	1	$283000
**Igloo annual Service Level Agreement (SLA)**	Annual	$10720	1	$10720
**Insta 360 Pro Camera**	2020	~$10000	1	$10000
**VR headsets**	2023	~$400	10	$4000
**VR Filming and editing – Simulation Technician salary for 1 week**	2022	$3150	1	$3150
**Simulated Patient (casual filming award rates)**	2022	$5000	1	$5000

* Not included in this table is the equipment used to edit video content, nor the maintenance costs of AHS.
**Costs in Australian Dollars (AUD)

Costs specific to Workshop design

Item description	Total cost (approx.)*
**Editing of LELAN video for viewing in 360° environment - Simulation Technician salary for 1 week**	$3150
**Simulation Educator faculty + Psychiatry faculty time (~40 hours) to design and pilot workshop**	$2800
**Simulated Patient time - pilot workshop**	$210
**Simulation Technician time – pilot workshop**	$350

*Costs in Australian Dollars (AUD)

Costs specific to Workshop delivery

Item description	Total cost (approx.) for each workshop*	Total cost (approx.) for workshops included in project (six)
**Simulated Patient time (5 hours per workshop)**	$210	$1260
**Simulation Technician time (6 hours per workshop)**	$350	$2100
**Psychiatrist facilitator time****	Variable	Variable

*Costs in Australian Dollars (AUD)
**Three Consultant Psychiatrists were present for each workshop. Some were paid as employees of the University of Adelaide, and others provided in-kind support as Titleholders of the University
